# *Salmonella enterica* serotypes causing infection in Kuwait during 2018–2021, determined by multi-locus sequence typing or whole genome sequencing

**DOI:** 10.1128/spectrum.02248-24

**Published:** 2025-04-09

**Authors:** Amani H. Al Fadhli, Wafaa Y. Jamal, Fatema Bibi Khodakhast, Glen P. Carter, Dieter Bulach, M. John Albert

**Affiliations:** 1 Department of Medical Laboratory Sciences, College of Allied Health Sciences, Kuwait University 718695, Jabriya, Hawalli Governate, Kuwait; 2 Department of Microbiology, College of Medicine, Kuwait University 62797, Jabriya, Hawalli Governate, Kuwait; 3 Department of Microbiology and Immunology, Peter Doherty Institute for Infection and Immunity, The University of Melbourne 198084, Parkville, Victoria, Australia; Rush University Medical Center, Chicago, Illinois, USA

**Keywords:** genomic surveillance, *Salmonella*, MLST, WGS, serotypes

## Abstract

Salmonellosis due to non-typhoidal *Salmonellae* (NTS) is a zoonotic infection that has epidemiological uniqueness in different settings. The current study aimed to determine the serotypes and the genetic diversity of human *Salmonella enterica* isolates causing infection in Kuwait. Isolates were obtained from feces of healthy adults and diarrheal patients between 2018 and 2021. Multi-locus sequence typing (MLST) was used to study sequence types (STs) and infer serotypes. Whole genome sequencing (WGS) was used to investigate six selected isolates, which included two isolates from a foodborne outbreak and two isolates whose serotypes could not be determined. Antibiotic susceptibility was studied by E-test and interpreted according to the Clinical and Laboratory Standards Institute guidelines. During the study period, 112/8,019 stool samples, 39/129,130 blood samples, 4/1,835 tissue samples, 3/1,209 pleural fluids, 3/9,388 pus samples, 4/80,799 urine samples, 1/7,053 endotracheal secretions, and 1/18 liver abscess samples were culture positive for *Salmonella*, yielding a total of 167 isolates with 30 different serotypes. *S*. Enteritidis (36.5%, *n* = 61), *S*. Typhimurium (14.97%, *n* = 25), *S*. Kentucky (5.9%, *n* = 10), and *S*. Newport (5.9%, *n* = 10) were the predominant serotypes. A new sequence type, ST 10217 corresponding to *S*. Schwarzengrund, was found by WGS. Two *S*. Enteritidis isolates from the foodborne outbreak showed a unique phylogenetic profile. In the phylogenetic analysis of serotypes, the number of clades was equal to the number of STs. No resistance to carbapenems was found among the isolates. This study provided data on the epidemiology of *Salmonella* serotypes causing infection in Kuwait.

**IMPORTANCE:**

Human salmonellosis due to nontyphoid *Salmonellae* is a major foodborne disease throughout the world. We determined the serotypes of isolates causing salmonellosis in Kuwait during the study period. We inferred the serotypes of isolates based on their sequence types as determined by multi-locus sequence typing, which is more amenable to laboratories than the traditional serotyping. By whole genome sequencing, we determined that the strain causing a foodborne outbreak was unique, and a new sequence type not in the serotyping scheme represented a rare serotype. We learnt the resistance pattern of isolates and lack of resistance to carbapenems that will be useful for treating multi-drug-resistant infection. Our data will contribute to planning strategies for treatment and control of salmonellosis and the epidemiology of salmonellosis in the Middle East.

## INTRODUCTION

*Salmonella enterica* is a common foodborne pathogen responsible for food poisoning in humans (1). Nontyphoidal salmonellosis causes an estimated 150 million cases of gastroenteritis worldwide annually, with approximately 60,000 deaths (2). *S. enterica* foodborne infections are primarily transmitted by poultry eggs and meat and their derived products (3). *S. enterica* is divided into six subspecies: *S. enterica* subsp. *enterica*, *S. enterica* subsp. *salamae*, *S. enterica* subsp. *arizonae*, *S. enterica* subsp. *diarizonae*, *S. enterica* subsp. *houtenae*, and *S. enterica* subsp. *indica* (4). Several serotypes of *S. enterica* subsp. *enterica* are responsible for most *Salmonella* infections in warm-blooded hosts, while other subspecies are isolated from the environment or cold-blooded hosts (5). Various *S. enterica* subsp. *enterica* serotypes can cause extra-intestinal infections, including *S*. Typhi and *S*. Paratyphi A, B, and C. Nontyphoidal *Salmonella*e (NTS) are other serotypes of the same subspecies, which usually infect a wide range of hosts and cause self-limiting gastroenteritis and extraintestinal infections. The two most widely distributed serotypes causing NTS infections are *S*. Typhimurium and *S*. Enteritidis. They spread from animals to humans via contaminated food products or by direct contact with infected animals or from human to human via contaminated hands (3, 6, 7).

Historically, serotyping was used to differentiate *S. enterica* isolates (8). Serotypes of *S. enterica* are often host-associated. Serotyping plays an important role in surveillance and outbreak investigation of salmonellosis (9, 10). The serotype/serovar classification of *S. enterica* isolates is based on the Kauffman–White scheme, which was first published in 1934 (11); so far, more than 2,610 serotypes have been documented worldwide (12).

Multi-locus-sequence typing (MLST) is a commonly used molecular method for differentiation in many bacterial species or genera (13). MLST data have been adapted for use for a range of applications, including analysis of evolutionary relationships (13–15), and the distribution of clonal isolates across different environments and hosts (16, 17). In the case of *S. enterica*, STs determined by MLST are correlated with serotypes (14, 18). Inferring serotype from ST provides a significant cost and time savings since serotyping is normally carried out at reference laboratories remote from the isolating laboratory (19). This approach is now increasingly used (8, 14, 19).

Whole genome sequencing (WGS) is now widely used in outbreak investigations. In some countries, WGS is used for the routine surveillance of foodborne diseases by public health laboratories (20). For reasons of cost, WGS was used in a limited way in the current study; primarily, it was used to enhance the analysis of a small group of isolates.

In Kuwait, *S. enterica* is a leading cause of diarrheal disease (21–23); despite this, there are scarce data on the distribution of serotypes (24). Clinical manifestation and epidemiology of *Salmonella* serotypes differ (25). The main aim of the present study was to determine the serotypes causing intestinal salmonellosis in Kuwait during 2018–2021 and their genetic diversity. In addition, we studied (i) *Salmonella* isolates related to a foodborne outbreak (compared the whole genome sequence of one selected pre-outbreak isolate and one post-outbreak isolate with those of two selected outbreak isolates to find out whether the outbreak isolate was unique), (ii) all isolates from extra-intestinal sites, as they were a small number, to cover all isolates during the study period, and (iii) the antibiotic susceptibility of all isolates according to their serotypes.

## MATERIALS AND METHODS

### *S. enterica* isolates

One hundred and sixty-seven *S. enterica* isolates (corresponding to a single isolate per patient from consecutive patients) were collected between 2018 and 2021 from inpatients and outpatients. The isolates originated from apparently healthy individuals who were screened by the General Medical Council of Kuwait for employment in the government and private sectors and patients who were treated at two government hospitals (Sheikh Jaber Al Ahmad and Mubarak Al Kabeer) and two polyclinics (Mahmoud Haji Haidar and Rumaithiyah). Among these 167 isolates, 40 originated from an outbreak of diarrhea that occurred in July 2018 (Table S1). This outbreak was linked to contaminated food served at an eatery. The affected patients sought care at various treatment centers, including Mubarak Al Kabeer Hospital. There was a noticeable surge in the number of diarrheal stools received at the clinical microbiology laboratory of Mubarak Al Kabeer Hospital during the outbreak (10–17 July 2018). The investigation of the outbreak was carried out by the Public Health Department of the State of Kuwait (unpublished). Other details of the investigation are not known to us. The occurrence of this outbreak was known before the isolates were selected for the current study.

### Culture method

Specimens included stool and extra-intestinal samples, such as blood, urine, pus, tissue, pleural fluid, endotracheal secretion, or liver abscess, for culture according to the diagnosis of a patient (for details see Tables S1 to S4). Isolates were stored at −80°C in CryoBank beads (Mast Group Limited, Merseyside, UK) until used for the study. The culture method for specimens from extra-intestinal sites was done as described previously (https://www.gov.uk/uk-standards-for-microbiology-investigations-smi-quality-and-consistency-in-clinical-laboratories). Stool was cultured on MacConkey agar (Oxoid, Basingstoke, UK) and *Salmonella–Shigella* agar (SS) (Thermo Fisher Scientific, Waltham, MA, USA). Stool was also inoculated into Selenite F broth (Becton-Dickinson, Franklin Lakes, NJ, USA) for the selective enrichment of *Salmonella*. The inoculated Selenite F broth was subcultured onto SS agar after incubation. Blood was cultured using blood culture bottle (Bactec 240, Becton-Dickinson) for up 5 days, and when the growth signal appeared, it was subcultured onto MacConkey and blood agars (Thermo Fisher Scientific). Urine was cultured using CLED (Thermo Fisher Scientific). Tissue, endotracheal secretion, pleural fluid, and pus were cultured on blood and MacConkey agars. All media were incubated at 37°C in normal air for 24 h. Subculturing of Selenite F broth was also done after 24 h of incubation. Suspected *Salmonella* colonies from agar media were selected for confirmation by matrix-assisted laser desorption/ionization time-of-flight mass spectrometry (bioMerieux, Marcy-l'Etoile, France). These included lactose nonfermenting pale colonies from MacConkey agar, lactose nonfermenting pale colonies with black center from SS agar, and colonies from blood agar.

### Antimicrobial susceptibility testing

Among the 167 isolates included in the study, 137 isolates were from 2018 to 2020 whose susceptibility was previously evaluated (23). In that study, the serogroups but not the serotypes were determined. In the current study, we grouped the isolates based on serotypes and reanalyzed the antibiogram as per serotype groups. In addition, we tested the susceptibility of 30 isolates from 2021. Susceptibility was performed using E-test (bioMerieux), and breakpoints were determined according to the interpretive criteria recommended by the Clinical and Laboratory Standards Institute (26); specifically, the breakpoints were: ampicillin (8 µg/mL), cefotaxime (1 µg/mL), chloramphenicol (8 µg/mL), ciprofloxacin (0.06 µg/mL), ertapenem (0.5 µg/mL), gentamicin (4 µg/mL), meropenem (1 µg/mL), and trimethoprim/sulfamethoxazole (2/38 µg/mL). *Escherichia coli* ATCC 25922 strain was used as a quality control strain in each batch of tests. Multidrug-resistant was defined as resistance to three or more different classes of antibiotics.

### DNA extraction

DNA was extracted by the boiling method (27) from all isolates for MLST. In brief, isolates were grown on MacConkey agar for 24 h at 37°C, and a few colonies were resuspended in 300 µL of deionized water in a tube. The suspension was heated to 95°C for 10 min in a water bath and immediately chilled on ice. The suspension was centrifuged at 13,000 × *g* for 5 min. The supernatant was used as the source of DNA. The purity of DNA was checked by the ratio of absorbance at 260/280 nm, which ranged between 1.8 and 1.9 (accepted value for purity). The yield of DNA ranged between 205 and 456 ng/µL. The DNA was stored in a sterile microtube and stored at −20°C until studied.

### MLST and phylogenetic tree

A detailed procedure for PCR-based MLST is described at the EnteroBase website (https://enterobase.readthedocs.io/en/latest). The housekeeping genes used for determination of ST were: *aroC*, *dnaN*, *hemD*, *hisD*, *purE*, *sucA*, and *thrA* (14). A combination of positive PCR results for certain housekeeping genes corresponds to a certain sequence type, which corresponds to a certain serotype. Many studies showed a complete correlation between the STs of *S. enterica* and serotypes. Therefore, by determining the ST of a *S. enterica* isolate, we can accurately infer its serotype. The serotype of an isolate was inferred from its ST using previously published tables (14). MEGA cluster analysis was used to construct the phylogenetic tree (28).

### Whole genome sequencing

A total of six isolates were subjected to WGS. These included four *S*. Enteritidis isolates—two from the outbreak (isolate no. 1107 cultured on 11 July 2018 and isolate no. 1121 cultured on 11 July 2018), one prior to the outbreak (isolate no. 1021 cultured on 21 May 2018), one after the cessation of the outbreak (no. 1158 cultured on 20 September 2021), and two *S*. Schwarzengrund isolates whose STs could not be determined (isolate no. 1024 cultured on 26 August 2018 and isolate no. 1069 cultured on 9 July 2019). The *S*. Enteritidis isolates were randomly selected based on the daily isolation rate in the stool. A determination was made when the outbreak started and ended (see below under Results).

For sequencing, genomic DNA was extracted from the isolates using the QIAmp Fast DNA Stool Mini Kit (Qiagen, Hilden, Germany). Sequencing libraries were prepared using the Nextera XT DNA Sample Preparation Kit (Illumina, CA, USA), and the sequence read data were produced on the Illumina NextSeq instrument (paired end, 150 base reads). The quality of raw reads was assessed by SeqKit (https://github.com/shenwei356/seqkit). Confirmation of isolate purity and the taxonomic classification (i.e., *S. enterica*) of each isolate were performed using Kraken2 (29) and Genome Taxonomy Database, respectively. Serotype classification was performed using Sistr and Seqsero 2 (30, 31). A draft genome sequence was assembled for each isolate using Spades v3.9 (32), and the genome sequence was annotated using Prokka v1.12b (33). Where an isolate had a new ST, the genome sequence was submitted to EnteroBase (https://enterobase.warwick.ac.uk/) for ST assignment. Comparative core genome phylogenetic analysis was performed using Bohra (https://github.com/MDU-PHL/bohra). The reference genome sequence used for the core genome comparison was from *S. enterica* subsp. *enterica* serotype Enteritidis str. P125109 (NCTC 13349 culture collection, sequence: NC_011294.1). Antibiotic resistance genes were detected using Abritamr (https://github.com/MDU-PHL/abritamr) and the National Database of Antibiotic Resistant Organisms database of genes (https://www.ncbi.nlm.nih.gov/pathogens/antimicrobial-resistance/).

### Regional and international contexts for the Kuwaiti *S*. Enteritidis isolates

The sequences of isolates as part of this study have been added to the NCBI Pathogen Detection Project (National Center for Biotechnology Information, National Institutes of Health, Bethesda, MD, USA, 2016 May) (accessed 10 December 2024; https://www.ncbi.nlm.nih.gov/pathogens). Isolate metadata used in the analysis are available at https://www.ncbi.nlm.nih.gov/pathogens/isolates/. Comparative core genome phylogenetic analysis was performed using Bohra (https://github.com/kristyhoran/bohra).

## RESULTS

### Bacterial isolates

The details of specimens from which isolates were obtained are shown in Table 1. One hundred and twelve (1.38%) stool cultures, 39 (0.03%) blood cultures, four (0.21%) tissue specimens, three (0.25%) pleural fluid samples, three (0.031%) pus samples, four (0.008%) urine samples, one (0.014%) endotracheal secretion sample, and one (5.55%) liver abscess sample were positive for *Salmonella*.

**TABLE 1:** Clinical samples from which *Salmonella* was isolated

Source	Year				Total no. of isolates/total no. of samples processed for isolation
	2018	2019	2020	2021	
Stool	75	21	1	15	112/8,091
Blood	15	14	4	6	39/129,130
Tissue	1	0	1	2	4/1,835
Pleural fluid	1	0	2	0	3/1,209
Pus	0	1	1	1	3/9,388
Urine	0	0	0	4	4/80,799
Endotracheal secretion	0	0	0	1	1/7,053
Liver abscess	0	0	0	1	1/18
Total	92	36	9	30	167/236,296

Among the isolates in Mubarak Al Kabeer Hospital were 40 isolates obtained during a foodborne outbreak caused by *S*. Enteritidis in July 2018 (Table S1). The details of isolates from stool, blood, and other extra-intestinal sites are shown in Tables S2 to S4, respectively. The details of the inferred serotypes of isolates from different hospitals and polyclinics are shown in Table S5.

The number of isolates studied from centers other than Mubarak Al Kabeer Hospital was small (*n* = 21). The total number of isolates available for the year 2020 was also small (*n* = 9). The serotypes of 165 isolates could be determined from their STs. However, the STs of two isolates, 1024 and 1069, could not be inferred from the existing scheme (14), and these isolates were subjected to WGS; a new ST was assigned by submitting the genome sequence to EnteroBase. Both isolates were of ST10217. Based on genomic relationship, these ST10217 isolates were inferred to be *S. enterica* serotype Schwarzengrund, and this classification was confirmed using Sistr, the *Salmonella* serotype prediction tool. These *S*. Schwarzengrund isolates originated from a specimen in Mubarak Al Kabeer Hospital and from another specimen obtained during screening of recruits for employment by the General Medical Council of Kuwait.

There were 146 isolates that originated from Mubarak Al Kabeer Hospital. They represented 26 different serotypes. The predominant serotypes in Mubarak Al Kabeer Hospital were *S*. Enteritidis (ST11) (*n* = 59), *S*. Typhimurium (ST19) (*n* = 23), *S*. Kentucky (ST198) (*n* = 10), *S*. Typhi (ST1 and ST2209) (*n* = 9) (we included this information on *S*. Typhi to get a complete picture of all *Salmonella* infections), *S*. Newport (ST158) (*n* = 6), and *S*. Agona (ST13) (*n* = 6). In Mubarak Al Kabeer Hospital, some serotypes were isolated for more than a 1 year period. These included: *S*. Agona, *S*. Bareilly, *S*. Enteritidis, *S*. Heidelberg, *S*. Infantis, *S*. Kentucky, *S*. Livingstone, *S*. Newport, *S*. Typhi, and *S*. Typhimurium.

There were other serotypes that were detected in low numbers. The serotypes*—S*. Enteritidis, *S*. Typhimurium, *S*. Heidelberg, *S*. Mbandaka, *S*. Livingstone, *S*. Baireilly, *S*. Braenderup, *S*. Newport, and *S*. Schwanzengrund—were isolated at more than one center (Table 2).

**TABLE 2:** Details of 167 *S*. *enterica* isolates studied according to serotype, sequence type (ST), and location

Serotype	No. of isolates	ST (no. of isolates)	Location (no. of isolates)
*S.* Enteritidis	61	58 (11), 183 (2), 1,918 (1)	MA^*a*^ (59), MH^*b*^ (1), SJ^*c*^ (1)
*S.* Typhimurium	25	19 (24), 29 (1)	MA (23), SJ (1), KG^*d*^ (1)
*S.* Kentucky	10	198 (10)	MA (10)
*S.* Newport	10	158 (9), 166 (1)	MA (6), KG (4)
*S.* Typhi	9	1 (8), 2,209 (1)	MA (9)
*S.* Agona	6	13 (6)	MA (6)
*S.* Mbandaka	5	413 (5)	MA (3), MH (1), KG (1)
*S*. Heidelberg	4	15 (4)	MA (3), KG (1)
*S.* Bareilly	4	203 (4)	MA (3), KG (1)
*S.* Braenderup	4	22 (4)	KG (3), MA (1)
*S.* Poona	4	308 (4)	MA (4)
*S.* Infantis	3	32 (3)	MA (3)
*S.* Livingston	3	543 (2), 457 (1)	MA (2), KG (1)
*S*. Schwarzengrund	3	10,217 (2), 2,488 (1)	KG (2), MA (1)
*S*. Hadar	2	33 (2)	MA (2)
*S*. Cubana	1	286 (1)	MH (1)
*S*. Alachua	1	1,298 (1)	SJ (1)
*S*. Anatum	1	64 (1)	MA (1)
*S*. Chester	1	2,063 (1)	MA (1)
*S*. Cotham	1	617 (1)	MA (1)
*S*. Grumpensis	1	751 (1)	MA (1)
*S*. Kedougou	1	1,543 (1)	MA (1)
*S*. Montevideo	1	138 (1)	MA (1)
*S*. Reading	1	44 (1)	MA (1)
*S*. Saintpaul	1	27 (1)	MA (1)
*S*. Sandiego	1	8,358 (1)	MA (1)
*S*. Seftenberg	1	14 (1)	MA (1)
*S*. Tennessee	1	319 (1)	MA (1)
*S*. Orion	1	639 (1)	RP^*e*^ (1)

^*a*^ Mubarak Al Kabeer Hospital.

^*b*^ Mahmoud Haji Haidar Polyclinic.

^*c*^ Sheikh Jaber Hospital.

^*d*^ Kuwait General Medical Council.

^*e*^ Rumaithiyah Polyclinic.

The details of 167 *S*. *enterica* isolates studied according to serotypes, location, and ST are shown in Table 2. *S*. Enteritidis, *S*. Livingstone, *S*. Newport, *S*. Typhi, *S*. Schwarzengrund, and *S*. Typhimurium were represented by more than one ST.

### Antimicrobial susceptibility testing

A high rate of resistance was found in *S*. Enteritidis to ciprofloxacin 78% (48/61); in *S*. Kentucky to ampicillin 60% (6/10), gentamicin 70% (7/10), and ciprofloxacin 90% (9/10); in *S*. Typhi to ciprofloxacin 66.7% (6/9); in *S*. Agona to ciprofloxacin 66.7% (4/6); in *S*. Bareilly to trimethoprim 50% (2/4) and ciprofloxacin 50% (2/4); in *S*. Braenderup to ciprofloxacin 50% (2/4); in *S*. Infantis to ciprofloxacin 66.7% (2/3); in *S*. Hadar to ampicillin 50% (1/2) and ciprofloxacin 100% (2/2); in *S*. Orion to ampicillin 100% (1/1) and cefotaxime 100% (1/1); and in *S*. Anatum to ciprofloxacin 100% (1/1). Several serotypes represented by single isolates were not resistant to any antibiotics. None of the serotypes were resistant to meropenem or ertapenem (Table 3).

**TABLE 3:** Susceptibility of 167 *Salmonella* isolates according to serotypes^*a*^

Serotype	No. of isolates	No. (%) of isolates resistant to antibiotic:					
		C	AM	TMP	GM	CTX	CIP
*S*. Enteritidis	61	0	4 (6.6)	1 (1.6)	0	0	48 (78.7)
*S.* Typhimurium	25	1 (4)	2 (8)	0	0	0	4(16)
*S*. Kentucky	10	1 (10)	6 (60)	1 (10)	7 (70)	0	9 (90)
*S*. Newport	10	1 (10)	1 (10)	1 (10)	1 (10)	0	1 (10)
*S*. Typhi	9	3 (33.3)	4 (44.4)	1 (11.1)	0	2 (22.2)	6 (66.7)
*S*. Agona	6	1 (16.7)	2 (33.3)	1 (16.7)	0	0	4 (66.7)
*S*. Mbandaka	5	1 (20)	1 (20)	0	0	0	0
*S*. Poona	4	0	0	0	0	0	1 (25)
*S*. Heidelberg	4	0	0	0	0	0	0
*S*. Bareilly	4	0	0	2 (50)	0	0	2 (50)
*S*. Braenderup	4	0	0	0	0	0	2 (50)
*S*. Livingstone	3	0	0	0	0	0	1 (33.3)
*S*. Schwarzengrund	3	0	0	0	0	0	1 (33.3)
*S.* Infantis	3	2 (66.7)	1 (33.3)	1 (33.3)	1 (33.3)	1 (33.3)	2 (66.7)
*S*. Hadar	2	0	1 (50)	0	0	0	2 (100)
*S*. Orion	1	0	1 (100)	0	0	1 (100)	0
*S*. Anatum	1	0	0	0	0	0	1 (100)

^*a*^ C: chloramphenicol, AM: ampicillin, TMP: trimethoprim, GM: gentamicin, CTX: cefotaxime, and CIP: ciprofloxacin. All isolates were susceptible to meropenem and ertapenem. *S*. Tennessee, *S*. Sandiego, *S*. Reading, *S*. Kedougou, *S.* Heidelberg, *S.* Chester, *S.* Cubana, *S.* Grumpensis, *S*. Alachua, *S*. Cotham, *S*. Montevideo, *S*. Saintpaul, and *S*. Senftenberg were all susceptible to the tested antimicrobial agents.

The resistance patterns of the two outbreak isolates (1107 and 1121), the pre-outbreak isolate (1021), and the post-outbreak isolate (1158) were singled out. Isolate 1021 was susceptible to all the eight antibiotics. The other three isolates were resistant to ciprofloxacin. These three isolates had a *gyrA* mutation (gyrA_A67P), while all isolates possessed the efflux pump genes, *mdsA* and *mdsB*.

### MLST and phylogenetic tree

We constructed dendrograms for *S*. Enteritidis, *S*. Typhimurium, *S*. Newport, and *S*. Typhi (predominant serotypes, which were represented by more than one ST). The housekeeping genes and the allele numbers are listed in Table S6. For the 61 isolates classified as *S*. Enteritidis, 58 isolates were ST11; two were ST183; and one was ST1918. The three STs of *S*. Enteritidis (ST11, ST183, and ST1918) were distributed into three clades. *S*. Typhimurium isolates (with ST19 and ST29), *S*. Newport isolates (with ST158 and ST166), and *S*. Typhi isolates (with ST1 and ST2209) were distributed into two clades each (Fig. S1).

### Core genome comparison

The core genomes of two *S*. Enteritidis isolates cultured during the outbreak (1107 and 1121) had no single nucleotide polymorphism (SNP) differences and were different from the pre-(1021) and post-outbreak isolates (1158). The pairwise SNP distances are shown in Table 4.

**TABLE 4:** Pairwise core genome SNP distances of *S*. *Enteritidis*

Isolate	1021^*a*^	1107^*b*^	1121^*c*^	1158^*d*^	P125109 (reference)
1021	0	91	91	108	69
1107	91	0	0	27	60
1121	91	0	0	27	60
1158	108	27	27	0	77
P125109 (reference)	69	60	60	77	0

^*a*^ Pre-outbreak isolate.

^*b*^ Outbreak isolate.

^*c*^ Outbreak isolate.

^*d*^ Post-outbreak isolate.

The phylogenetic tree showed that the outbreak isolates were identical and different from the pre-and post-outbreak isolates (tree data not shown).

### Related isolate analysis of *S*. Enteritidis

Highly related isolates in the pathogen detection (PD) collection of isolates (700,242 *S*. Enteritidis isolates as of 10 December 2024) are grouped by the PD cluster identifier (Fig. S2). Among the four *S*. Enteritidis isolates, three Kuwait isolates have been assigned to PDS000026888.164 (both outbreak and post-outbreak isolates, see Table S7), and one isolate has been assigned to PD cluster PDS000026860.246. Among the 694 PDS000026888.164 isolates with collection dates ranging from 2007 to 2024, most isolates were collected in Saudi Arabia (*n* = 391) and the UK (*n* = 343), China (*n* = 96) and the USA (*n* = 39) with a small number of isolates from Australia (*n* = 8), South Africa (*n* = 1), the Netherlands (*n* = 3), Lebanon (*n* = 1), Kuwait (*n* = 5), Iraq (*n* = 2), and Canada (*n* = 2). Note that two isolates had no location information. The outbreak isolates (1107 and 1121) were nearly identical to an isolate from the USA (GCA_016289335). For the 873 PDS000026860.246 isolates (includes Kuwait isolate 1021; see Table S7), collection dates ranged from 2004 to 2024. Most isolates were collected in the UK (*n* = 663), Saudi Arabia (*n* = 17) and USA (*n* = 58) with 86 isolates from other countries in Europe, North America (*n* = 3), and Oceania (*n* = 20). Twenty-four isolates had no location noted. None of the isolates were identical to the 1021 isolate.

The sequences of the above four *S*. Enteritidis isolates published in the Bioproject PRJNA929096 have now been analyzed by NCBI and included in the NCBI pathogen detection collection (https://www.ncbi.nlm.nih.gov/pathogens) with nearly 600,000 *S*. *enterica* genomes available (accessed on 17 February 2024). Three isolates have been classified in the PDG000000002.2917/PD000026888.122 SNP cluster. The two outbreak isolates (isolates 1121 and 1107) were paired. The post-outbreak isolate (isolate 1158) was highly related to the outbreak isolates (being included in the same cluster as the outbreak isolates).

We discovered a novel ST10217 of *S. enterica* that was inferred to be serotype *S*. Schwarzengrund based on genomic relatedness to other *S*. Schwarzengrund genome sequences. To identify related international isolates, we searched https://www.ncbi.nlm.nih.gov/pathogens (accessed on 23 January 2023) for *S. enterica* isolates classified as ‘Schwarzengrund.’ It revealed 2,206 isolates with draft sequences. By using Mashtree, we were able to determine the genomic relationship among the isolates (Fig. S3A). It was interesting to note that our isolates were most related to isolates from the US and the UK (Fig. S3B). A group of isolates was listed in the same “SNP cluster” of SNP: PDS000001366.18 (SNP clusters were assigned at https://www.ncbi.nlm.nih.gov/pathogens/).

## DISCUSSION

NTS are the most common pathogens associated with diarrhea in Kuwait (24). *S*. Enteritidis was the most prevalent serotype associated with nontyphoid salmonellosis in Kuwait, which is in line with the global trend reported by the US Centers for Disease Control and Prevention (6) and EU Food Safety Authority (34).

In the EnteroBase database, ST11 accounted for 81% of the 62,152 entries for *S*. Enteritidis (http://enterobase.warwick.ac.uk.) (accessed on 25 June 2023). Globally, ST11 represents the most prevalent ST among *S*. Enteritidis (94.8%) (35). In line with this, we found that most of our isolates belonged to ST11. The success of ST11 as a pathogen may be related to its ability to survive in different environments, including humans, food items, and poultry slaughterhouses (36). It was interesting to observe that one of our *S*. Enteritidis isolates belonged to ST183. In the EnteroBase database, the 1,721 isolates identified as ST183 (as of 1 Oct. 2023) mostly originated in the United Kingdom, France, and Germany (37), suggesting possible importation of this ST from Europe. We also identified *S*. Enteritidis ST1918, a serotype that had not been previously detected in Kuwait (24). There is not much information on the distribution of this ST.

*S*. Typhimurium, which included the ST19 isolates, was the second most common serotype isolated in this study. This is an international lineage that most often causes serious disease and death (38, 39). *S*. Newport was the third most common serotype causing infection. This finding aligns with a previous report from the USA (40). Most of the isolates belonged to ST158. *S*. Kentucky (ST198) is a polyphyletic serotype with no common ancestor among the various STs. This serotype has different ecologies, host characteristics, and geographic distribution (41, 42). Most of our *S*. Typhi isolates belonged to ST1, which is consistent with a previous study (43). ST1 has been highly successful in global dissemination (43). We included the information on *S*. Typhi with that of nontyphoidal *Salmonella* to get a complete picture of *Salmonella* infection in our study. Our *S*. Agona isolates belonged to ST13, whose origin is likely to be poultry (44).

Meropenem is the preferred antibiotic for treating multidrug-resistant *Salmonella* infections when cephalosporins and quinolones are ineffective. Based on our study, this remains the drug of choice in Kuwait since none of our isolates were resistant to meropenem or ertapenem. It should, however, be noted that carbapenem resistance rates are increasing, with several studies having reported the isolation of carbapenem-resistant *Salmonella* previously in Pakistan, Taiwan, and China (45–47). This highlights the ongoing need for appropriate local surveillance for antimicrobial resistance screening activities in Kuwait. A large proportion of our *S. enterica* isolates were resistant to ciprofloxacin. This is concordant with previous reports on clinical isolates from Kuwait (23). The resistance rate of *S*. Typhimurium to ciprofloxacin was relatively high in our study compared to reports from China (47), Belgium (48), and Saudi Arabia (49). Resistance to cefotaxime was present in one-fifth of our *S*. Typhi isolates, whereas it was absent in isolates in Belgium (48) and Saudi Arabia (49). However, the ampicillin resistance rate in our *S*. Typhimurium isolates was relatively low compared to a high rate of resistance in other countries, including China (47), Italy (50), and Belgium (48). The high rate of resistance of *S*. Kentucky to ampicillin, ciprofloxacin, and gentamicin in our study was like that from France (42), suggesting that cefotaxime might be a better empiric antibiotic for treatment than ciprofloxacin. The prevalence of resistance to ciprofloxacin in our *S*. Enteritidis isolates was higher than that in China (47) and Belgium (48) and indicates a limited therapeutic option for treatment of this serotype. Resistance to ampicillin in our *S*. Newport isolates was relatively low compared to Italian isolates (50). The high rate of resistance to ampicillin and ciprofloxacin in our *S*. Infantis isolates was like that from Italy (51). However, resistance to chloramphenicol was not detected in the study from Italy compared to our study (51).

There were no core genome differences in the two *S*. Enteritidis outbreak isolates that were sequenced, consistent with these isolates being from a common origin. In addition, there were significant SNP differences in pre- and post-outbreak isolates. The clustering of isolates in Fig. S2 showed that the two outbreak isolates were paired and likely to be localized to Kuwait. The post-outbreak isolate, 1158, which was included in the same cluster as the outbreak isolates, appeared to be from another source because of the intervening isolates in the tree from a variety of international sources. Overview of the relationship of the NCBI Pathogen Detection Genomes suggested the relationship of Kuwait *S*. Enteritidis isolates with the isolates from other parts of the world. Of note is the relationship with isolates from nearby countries of Saudi Arabia, Iraq, and Lebanon. The existence of only one isolate from the USA identical to the 1107 and 1158 outbreak isolates suggested that the outbreak strain might have been widely distributed, although it was not possible to identify the origin of the strain. The dominance of isolates from a small number of countries indicated a sampling bias that meant that the genetic diversity and the geographic distribution of strains in the clusters were not adequately covered.

The two outbreak isolates (1107 and 1121), pre-outbreak isolate (1021), and post-outbreak isolate (1158) carried the efflux pump genes, *mdsA* and *mdsB*. The* mdsAB* gene mediates both virulence and drug resistance (52). The three isolates that were ciprofloxacin-resistant carried the *gyrA* mutation (gyrA_A67P) responsible for resistance (53).

We discovered a novel ST10217 of *S. enterica* that was inferred to be serotype *S*. Schwarzengrund. This serotype was related to serotypes from the US and the UK. Continuous monitoring of serotypes in Kuwait will reveal the extent of infection due to this novel ST.

Our findings on the serotype distribution of *S. enterica* infection in Kuwait contribute to the global data on salmonellosis. This epidemiological information can be used to track global clones and devise prevention strategies.

A limitation of the study is that a small number of isolates were available in 2020. This was related to the coronavirus disease 2019 pandemic restrictions on eateries remaining open and agrees with a report from the European Surveillance System that showed that salmonellosis cases in 2020 were significantly lower than in previous years (54). We could not sequence more isolates because of financial resource constraints. WGS of all the isolates in our study would have yielded better information.

### Conclusions

Our study has demonstrated the feasibility of utilizing STs to determine the serotypes of *Salmonella*. Antibiotic susceptibility testing showed the extent of resistance in *Salmonella* isolates in Kuwait. The WGS of selected isolates showed the existence of novel STs (hence a new serotype) and the uniqueness of the outbreak isolates. This study provides a valuable foundation for the start of a *Salmonella* surveillance in Kuwait.

## Data Availability

Raw reads of sequenced isolates of our study have been deposited in the National Center for Biotechnology Information (NCBI) under BioProject accession numbers PRJNA929096 (for *S*. Enteritidis isolates) and PRJNA934672 (for *S*. Schwarzengrund isolates). All relevant data are contained within the article and its supplemental material.

## SUPPLEMENTAL MATERIAL

Fig. S1 spectrum.02248-24-s0001.tif UPGMA dendrogram (magnifiable) from the pattern of pairwise differences in alleles that revealed the genetic relationships of STs among the *S. enterica* isolates, along with serotypes and year of isolation.

Fig. S2 spectrum.02248-24-s0002.tif An SNP distance tree (magnifiable) showing those isolates most related to the 2021 *S.* Enteritidis outbreak in Kuwait; these related isolates are classified as cluster PDS000026888.164 by NCBI.

Fig. S3A spectrum.02248-24-s0003.tif FIG S3A. A tree (magnifiable) showing the relationship between *S.* Schwarzengrund genome sequences (*n* = 2,206) available at NCBI (on 23 January 2023) and isolates 1024 and 1069 from Kuwait.

Fig. S3B spectrum.02248-24-s0004.tiff Inset of a portion of Figure 3A that contains isolates 1024 and 1069 from Kuwait.

Supplemental material spectrum.02248-24-s0005.docx Legends for all supplemental figures, as well as tables.

Supplemental tables spectrum.02248-24-s0006.docx Tables S1 to S5; Table S7.

Table S6 spectrum.02248-24-s0007.xlsx Alleles of housekeeping genes of *Salmonella* for MLST and determination of STs and serotypes.

ASM does not own the copyrights to Supplemental Material that may be linked to, or accessed through, an article. The authors have granted ASM a non-exclusive, world-wide license to publish the Supplemental Material files. Please contact the corresponding author directly for reuse.
