# Supplementary figures and images for: Salmonella enterica serotypes causing infection in Kuwait during 2018–2021, determined by multi-locus sequence typing or whole genome sequencing
Source: Microbiol Spectr. 2025 Apr 9;13(5):e02248-24. doi: 10.1128/spectrum.02248-24 (PMC12054093; doi:10.1128/spectrum.02248-24)

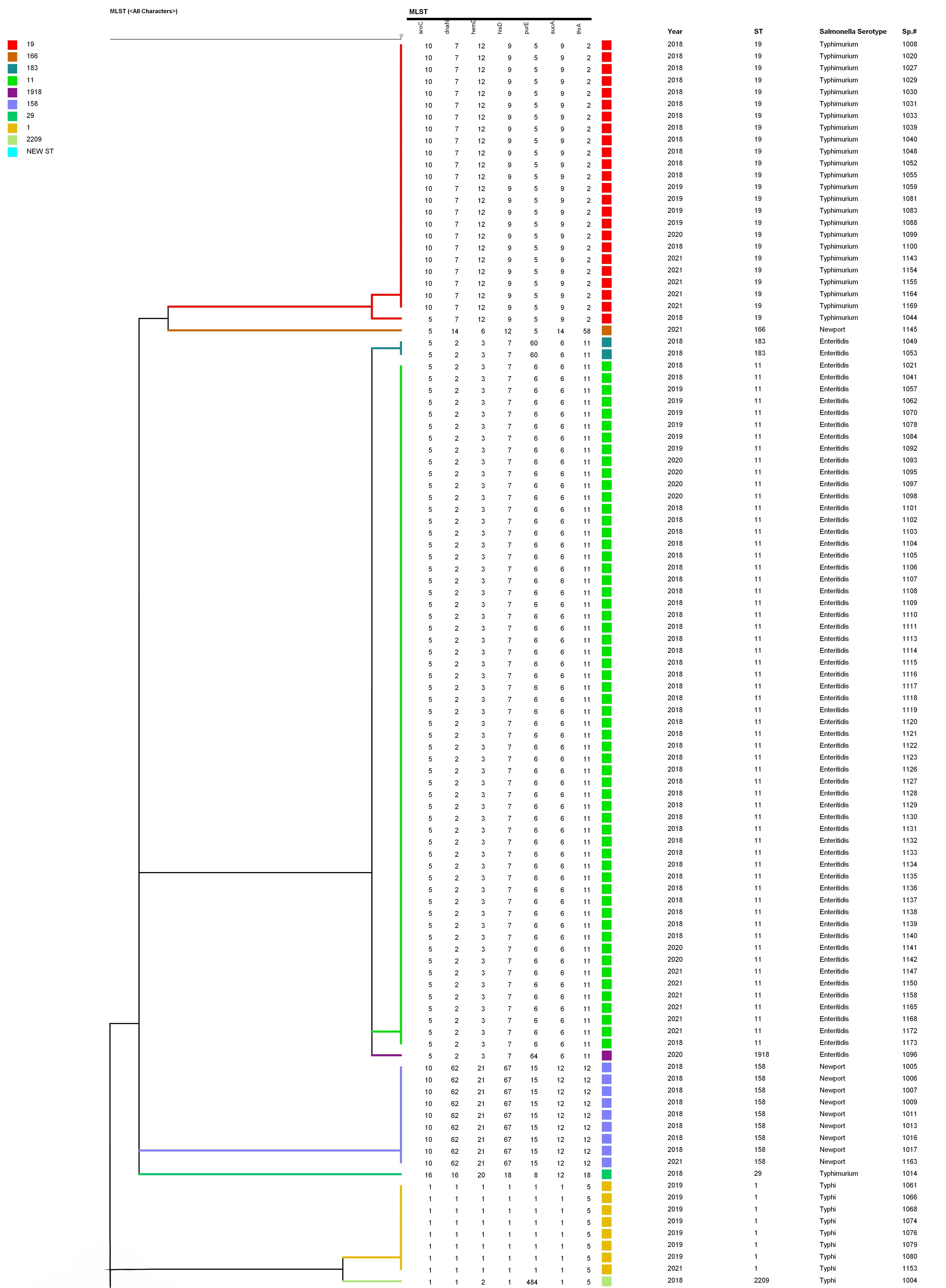

Supplement: Fig. S1 — UPGMA dendrogram (magnifiable) from the pattern of pairwise differences in alleles that revealed the genetic relationships of STs among the S. enterica isolates, along with serotypes and year of isolation. [file spectrum.02248-24-s0001.tif]

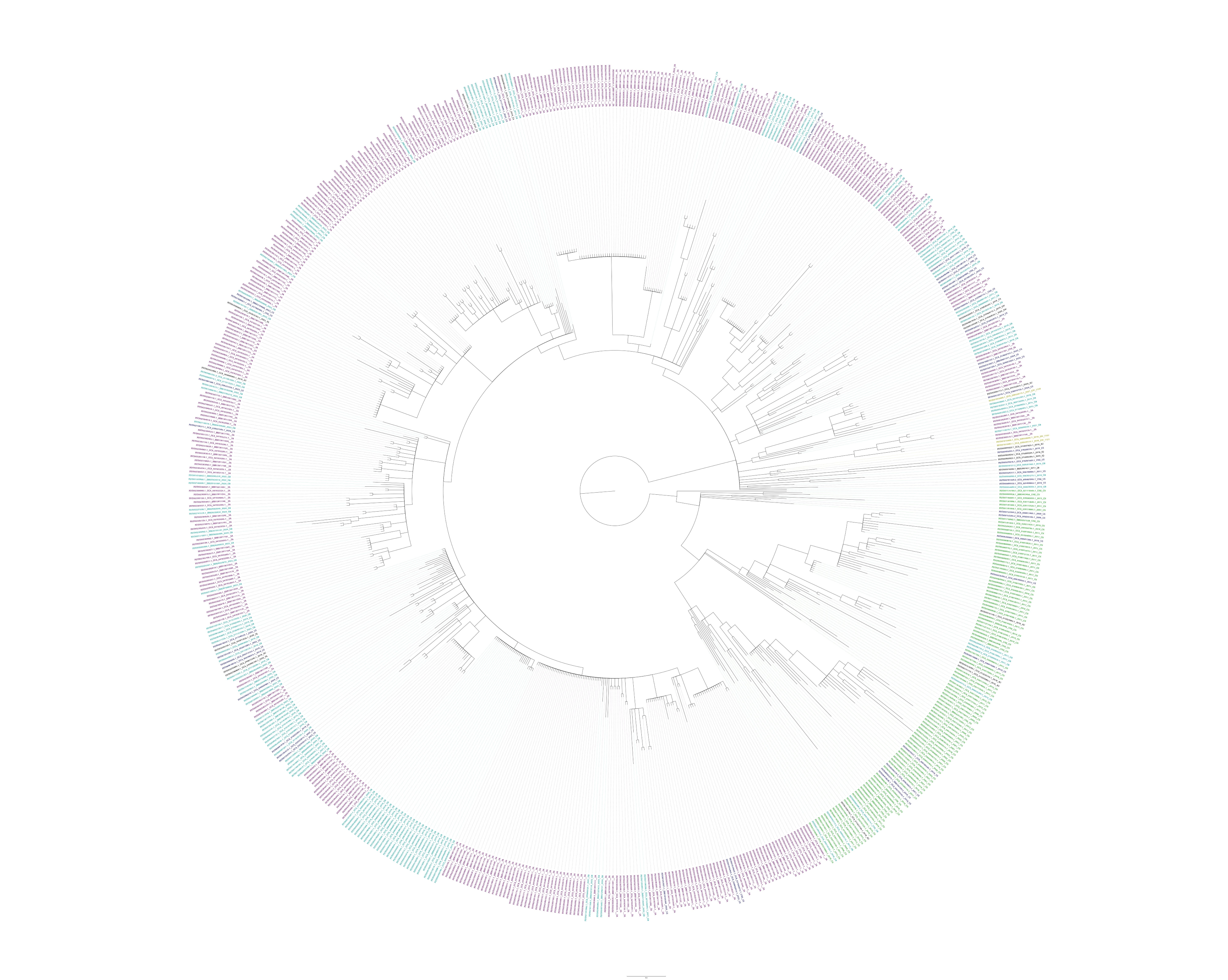

Supplement: Fig. S2 — An SNP distance tree (magnifiable) showing those isolates most related to the 2021 S. Enteritidis outbreak in Kuwait; these related isolates are classified as cluster PDS000026888.164 by NCBI. [file spectrum.02248-24-s0002.tif]

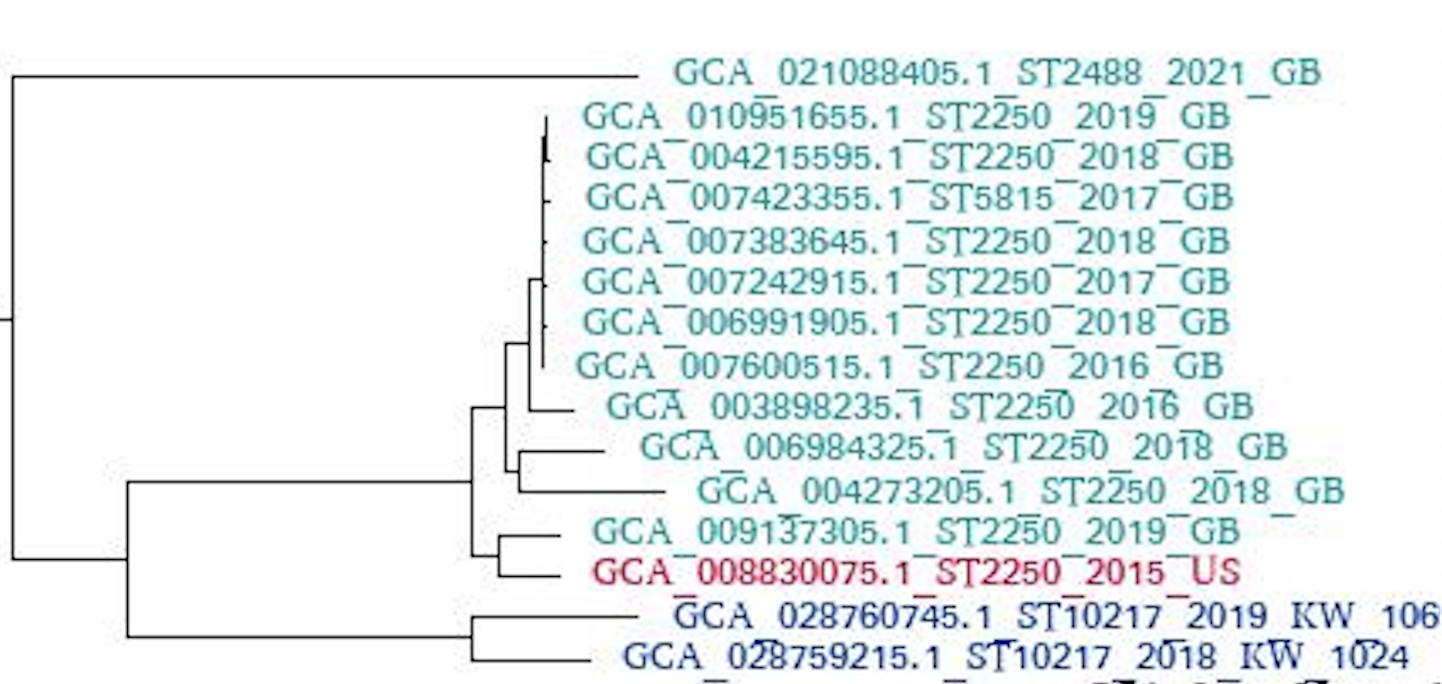

Supplement: Fig. S3B — Inset of a portion of Figure 3A that contains isolates 1024 and 1069 from Kuwait. [file spectrum.02248-24-s0004.tiff]
