# Supplementary material for: Salmonella enterica serotypes causing infection in Kuwait during 2018–2021, determined by multi-locus sequence typing or whole genome sequencing
Source: Microbiol Spectr. 2025 Apr 9;13(5):e02248-24. doi: 10.1128/spectrum.02248-24 (PMC12054093; doi:10.1128/spectrum.02248-24)
Supplement: Supplemental material — Legends for all supplemental figures, as well as tables. [file spectrum.02248-24-s0005.docx]

Supplemental legends

Table S1. The details of 40 *S. enterica* isolates cultured during the diarrheal outbreak in 2018 at Mubarak Al Kabeer Hospital.

Table S2. Distribution of *S. enterica* isolates from stool samples in different years in Kuwait.

Table S3. Distribution of *S. enterica* isolates from blood samples in different years in Kuwait.

Table S4. Distribution of *S. enterica* isolates from other samples in different years in Kuwait.

Table S5. The details of 167 *S. enterica* isolates studied from different years in Kuwait.

Table S6. Housekeeping genes and their allele numbers for salmonella MLST.

Table S7. Pathogen Detection (PD) cluster assignment for each of the four *S*. Enteritidis isolates.

FIG S1. UPGMA dendrogram (magnifiable) from the pattern of pairwise differences in alleles that revealed the genetic relationships of STs among the *S. enterica* isolates, along with serotypes and year of isolation.

FIG S2. A SNP distance tree (magnifiable) showing those isolates most related 2021 *S.* Enteritidis outbreak in Kuwait; these related isolates are classified as cluster PDS000026888.164 by NCBI. The SNP distance tree for cluster PDS000026888.164 exported from <https://www.ncbi.nlm.nih.gov/pathogens>/ on 10 December 2024. The scale bar shows SNP distance. This cluster contains three Kuwait isolates that have olive-colored taxon labels. The two outbreak isolates, PDT001610467.1 (isolate 1121) and PDT001610469.1 (isolate 1107) differ by 2 SNPs and these two isolates have a SNP distance of 27 SNPs from the post-outbreak isolate, PDT001610468.1 (isolate 1158). The pre-outbreak isolate (isolate 1021) was classified in a different cluster and therefore not shown in this tree. Taxon labels show the Pathogen Detection accession, the assembly accession, year of isolation and country of isolation (where available). The ISO 3166-1 alpha-2 code is used to abbreviate country names in the taxon labels. Taxon labels for GB are colored aqua, SA are colored purple, CN are colored green.

FIG S3A. A tree (magnifiable) showing the relationship between *S.* Schwarzengrund genome sequences (n=2206) available at NCBI (on 23 January 2023) and isolates 1024 and 1069 from Kuwait. The tree was inferred using Mashtree (<http://github.com/Iskatz/mashtree>). Taxon labels show the assembly accession, sequence type (ST), year of isolation and country of isolation (where available). The ISO 3166-1 alpha-2 code is used to abbreviate country names in the taxon labels. Taxon labels for US are colored red, GB are colored aqua, and KW are colored dark blue.

FIG S3B. Inset of a portion of Figure 3A that contains isolates 1024 and 1069 from Kuwait. The Kuwait *S.* Schwarzengrund isolates are most related to ST2250 isolates from the GB and US.
