# Supplementary material for: Salmonella enterica serotypes causing infection in Kuwait during 2018–2021, determined by multi-locus sequence typing or whole genome sequencing
Source: Microbiol Spectr. 2025 Apr 9;13(5):e02248-24. doi: 10.1128/spectrum.02248-24 (PMC12054093; doi:10.1128/spectrum.02248-24)
Supplement: Supplemental tables — Tables S1 to S5; Table S7. [file spectrum.02248-24-s0006.docx]

**S1 Table.** The details of 40 *S. enterica* isolates cultured during the diarrheal outbreak in 2018 at Mubarak Al Kabeer Hospital

| **Isolate no.** | **Patient gender** | **Patient age in year** | **Source** | **Sequence Type** | **Serotype** |
| --- | --- | --- | --- | --- | --- |
| 1100 | M | 48 | Stool | 19 | Typhimurium |
| 1101 | M | 27 | Blood | 11 | Enteritidis |
| 1102 | M | 27 | Blood | 11 | Enteritidis |
| 1103 | M | 10 | Stool | 11 | Enteritidis |
| 1104 | F | 12 | Stool | 11 | Enteritidis |
| 1105 | M | 2 | Stool | 11 | Enteritidis |
| 1106 | M | 33 | Stool | 11 | Enteritidis |
| 1107 | F | 5 | Stool | 11 | Enteritidis |
| 1108 | M | 65 | Stool | 11 | Enteritidis |
| 1109 | M | 2 | Stool | 11 | Enteritidis |
| 1110 | M | 7 | Stool | 11 | Enteritidis |
| 1111 | M | 6 | Stool | 11 | Enteritidis |
| 1112 | M | 5 | Stool | 308 | Poona |
| 1113 | M | 7 | Stool | 11 | Enteritidis |
| 1114 | F | 8 | Stool | 11 | Enteritidis |
| 1115 | M | 43 | Stool | 11 | Enteritidis |
| 1116 | M | 27 | Blood | 11 | Enteritidis |
| 1117 | M | 7 | Stool | 11 | Enteritidis |
| 1118 | F | 8 | Stool | 11 | Enteritidis |
| 1119 | F | 10 | Stool | 11 | Enteritidis |
| 1120 | M | 10 | Stool | 11 | Enteritidis |
| 1121 | F | 3 | Stool | 11 | Enteritidis |
| 1122 | M | 22 | Stool | 11 | Enteritidis |
| 1123 | M | 3 | Stool | 11 | Enteritidis |
| 1126 | M | 27 | Stool | 11 | Enteritidis |
| 1127 | F | 1 | Stool | 11 | Enteritidis |
| 1128 | M | 6 | Stool | 11 | Enteritidis |
| 1129 | F | 15 | Stool | 11 | Enteritidis |
| 1130 | M | 2 | Stool | 11 | Enteritidis |
| 1131 | F | 7 | Stool | 11 | Enteritidis |
| 1132 | M | 33 | Stool | 11 | Enteritidis |
| 1133 | M | 39 | Stool | 11 | Enteritidis |
| 1134 | F | 11 | Stool | 11 | Enteritidis |
| 1135 | F | 3 | Stool | 11 | Enteritidis |
| 1136 | M | 18 | Stool | 11 | Enteritidis |
| 1137 | F | 7 | Stool | 11 | Enteritidis |
| 1138 | M | 3 | Stool | 11 | Enteritidis |
| 1139 | M | 7 | Stool | 11 | Enteritidis |
| 1140 | M | 11 | Stool | 11 | Enteritidis |
| 1173 | M | 7 | Blood | 11 | Enteritidis |

F, female; M, male

| **Clinical specimen / Serotype** | **2018** | **2019** | **2020** | **2021** | **Total** |
| --- | --- | --- | --- | --- | --- |
| Stool |  |  |  |  | 112 |
| *S*. Enteritidis | 36 | 2 | 1 | 3 | 42 |
| *S*. Typhimurium | 12 | 3 |  | 1 | 16 |
| *S*. Kentucky | 3 | 2 |  | 4 | 9 |
| *S*. Newport | 8 |  |  | 1 | 9 |
| *S*. Agona | 2 | 1 |  |  | 3 |
| *S*. Mbandaka | 2 | 1 |  | 1 | 4 |
| *S*. Bareilly | 1 | 1 |  | 1 | 3 |
| *S*. Braenderup | 1 | 3 |  |  | 4 |
| *S*. Heidelberg |  | 2 |  | 1 | 3 |
| *S*. Poona | 1 |  |  |  | 1 |
| *S*. Infantis |  | 1 |  | 1 | 2 |
| *S*. Livingstone | 2 |  |  |  | 2 |
| *S*. Hadar | 2 |  |  |  | 2 |
| *S*. Schwarzengrund (novel ST) | 1 | 1 |  |  | 2 |
| *S*. Alachua |  | 1 |  |  | 1 |
| *S*. Anatum |  | 1 |  |  | 1 |
| *S*. Chester |  | 1 |  |  | 1 |
| *S*. Cubana |  | 1 |  |  | 1 |
| *S*. Kedougou | 1 |  |  |  | 1 |
| *S*. Montevideo |  |  |  | 1 | 1 |
| *S*. Orion | 1 |  |  |  | 1 |
| *S*. Senftenberg |  |  |  | 1 | 1 |
| *S*. Schwarzengrund | 1 |  |  |  | 1 |
| *S*. Tennessee | 1 |  |  |  | 1 |

**S2 Table.** Distribution of *S. enterica* isolates from stool culture from different years in Kuwait

| **Clinical specimen / Serotype** | **2018** | **2019** | **2020** | **2021** | **Total** |
| --- | --- | --- | --- | --- | --- |
| Blood |  |  |  |  | 39 |
| *S*. Enteritidis | 6 | 4 | 3 | 2 | 15 |
| *S*. Typhimurium | 2 | 1 |  | 1 | 4 |
| *S*. Typhi | 1 | 7 |  | 1 | 9 |
| *S*. Agona | 1 |  |  |  | 1 |
| *S*. Bareilly |  |  |  | 1 | 1 |
| *S.* Poona | 3 |  |  |  | 3 |
| *S*. Infantis |  |  |  | 1 | 1 |
| *S*. Livingstone |  | 1 |  |  | 1 |
| *S*. Cotham |  |  | 1 |  | **1** |
| *S*. Grumpensis |  | 1 |  |  | 1 |
| *S*. Reading | 1 |  |  |  | 1 |
| *S*. Sandiego | 1 |  |  |  | 1 |
|  |  |  |  |  |  |

**S3 Table**. Distribution of *S. enterica* isolates from blood culture years in Kuwait

| **Clinical specimen / Serotype** | **2018** | **2019** | **2020** | **2021** | **Total** |
| --- | --- | --- | --- | --- | --- |
| Blood |  |  |  |  | 39 |
| *S*. Enteritidis | 6 | 4 | 3 | 2 | 15 |
| *S*. Typhimurium | 2 | 1 |  | 1 | 4 |
| *S*. Typhi | 1 | 7 |  | 1 | 9 |
| *S*. Agona | 1 |  |  |  | 1 |
| *S*. Bareilly |  |  |  | 1 | 1 |
| *S.* Poona | 3 |  |  |  | 3 |
| *S*. Infantis |  |  |  | 1 | 1 |
| *S*. Livingstone |  | 1 |  |  | 1 |
| *S*. Cotham |  |  | 1 |  | **1** |
| *S*. Grumpensis |  | 1 |  |  | 1 |
| *S*. Reading | 1 |  |  |  | 1 |
| *S*. Sandiego | 1 |  |  |  | 1 |
|  |  |  |  |  |  |

**S4 Table**. Distribution of *S*. enterica isolates from other cultures from different years in Kuwait

**S5 Table.** The details of 167 *S. enterica* isolates studied from different years in Kuwait.

| **Location / Serotype** | **Number of the isolates in the indicated year** | | | | **Total no. of isolates** |
| --- | --- | --- | --- | --- | --- |
|  | **2018** | **2019** | **2020** | **2021** |  |
| Mahmoud Haji Haidar polyclinic | 1 | 3 |  |  | 4 |
| *S*. Eeritidisnt | 1 |  |  |  | 1 |
| *S*. Heidelberg |  | 1 |  |  | 1 |
| *S*. Cubana |  | 1 |  |  | 1 |
| *S*. Mbandaka |  | 1 |  |  | 1 |
| Sheikh Jaber Al Ahmad Hospital |  | 3 |  |  | 3 |
| *S*. Alachua |  | 1 |  |  | 1 |
| *S*. Enteritidis |  | 1 |  |  | 1 |
| *S*. Typhimurium |  | 1 |  |  | 1 |
| Mubarak Al Kabeer Hospital | 81 | 26 | 9 | 30 | 146 |
| *S*. Agona | 4 | 1 |  | 1 | 6 |
| *S*. Anatum |  | 1 |  |  | 1 |
| *S*. Bareilly | 1 |  |  | 2 | 3 |
| *S*. Braenderup | 1 |  |  |  | 1 |
| *S*. Chester |  | 1 |  |  | 1 |
| *S*. Cotham |  |  | 1 |  | 1 |
| *S*. Enteritidis | 41 | 5 | 7 | 6 | 59 |
| *S*. Grumpensis |  | 1 |  |  | 1 |
| *S*. Hadar | 2 |  |  |  | 2 |
| *S*. Heidelberg |  | 2 |  | 1 | 3 |
| *S*. Infantis |  | 1 |  | 2 | 3 |
| *S*. Kedougou | 1 |  |  |  | 1 |
| *S*. Kentucky | 3 | 2 |  | 5 | 10 |
| *S*. Livingstone | 1 | 1 |  |  | 2 |
| *S*. Mbandaka | 1 |  |  | 2 | 3 |
| *S.* Montevideo |  |  |  | 1 | 1 |
| *S*. Newport | 4 |  |  | 2 | 6 |
| *S*. Poona | 4 |  |  |  | 4 |
| *S*. Reading | 1 |  |  |  | 1 |
| *S*. Saintpaul |  |  |  | 1 | 1 |
| *S*. Sandiego | 1 |  |  |  | 1 |
| *S*. Senftenberg |  |  |  | 1 | 1 |
| *S*. Tennessee | 1 |  |  |  | 1 |
| *S*. Typhi | 1 | 7 |  | 1 | 9 |
| *S*. Typhimurium | 14 | 3 | 1 | 5 | 23 |
| *S.* Schwarzengrund (novel ST) |  | 1 |  |  | 1 |
| Kuwait General Medical Council | 9 | 4 |  |  | 13 |
| *S*. Bareilly |  | 1 |  |  | 1 |
| *S*. Braenderup |  | 3 |  |  | 3 |
| *S*. Livingstone | 1 |  |  |  | 1 |
| *S*. Mbandaka | 1 |  |  |  | 1 |
| *S*. Newport | 4 |  |  |  | 4 |
| *S*. Schwarzengrund | 1 |  |  |  | 1 |
| *S*. Typhimurium | 1 |  |  |  | 1 |
| *S*. Schwarzengrund (Novel ST) | 1 |  |  |  | 1 |
| Rumaithiyah polyclinic | 1 |  |  |  | 1 |
| *S*. Orion | 1 |  |  |  | 1 |
| Total | 92 | 36 | 9 | 30 | 167 |

**S7 Table**. Pathogen Detection (PD) cluster assignment for each of the four *S.* Enteritidis isolates

| **Isolate** | **PD cluster** | **Biosample** | **Assembly** | **Run** |
| --- | --- | --- | --- | --- |
| 1121 | PDS000026888.164 | SAMN32951888 | GCA_028559175.1 | SRR23264845 |
| 1158 | PDS000026888.164 | SAMN32951887 | GCA_028558775.1 | SRR23264846 |
| 1107 | PDS000026888.164 | SAMN32951886 | GCA_028558695.1 | SRR23264847 |
| 1021 | PDS000026860.246 | SAMN32951885 | GCA_028558705.1 | SRR23264848 |
